# Case Report: Successful rechallenge of cadonilimab-induced cystitis, adrenal insufficiency, and diabetic ketoacidosis in a patient with metastatic gastric-type endocervical adenocarcinoma

**DOI:** 10.3389/fonc.2026.1836563

**Published:** 2026-05-20

**Authors:** Zhan Peng, JiaJin Yang, Huan Li, WuGang Lei, HaiYan Yang, XueWen Liu

**Affiliations:** 1 Department of Obstetrics and Gynecology, Fengcheng People’s Hospital, Yichun University, Yichun, Jiangxi, China; 2 Department of Oncology, Fengcheng People’s Hospital, Yichun University, Yichun, Jiangxi, China; 3 Department of Obstetrics and Gynecology, Fengcheng Maternal and Child Health Hospital, Yichun, Jiangxi, China; 4 Department of Radiology, Fengcheng People’s Hospital, Yichun University, Yichun, Jiangxi, China; 5 Department of Endocrinology and Metabolism, Fengcheng People’s Hospital, Yichun University, Yichun, Jiangxi, China; 6 Department of Oncology, Ganzhou Hospital-Nanfang Hospital, Southern Medical University(Ganzhou People’s Hospital), Ganzhou, Jiangxi, China; 7 Ganzhou Municipal Key Laboratory of Tumor Radiobiology, Ganzhou People’s Hospital, Ganzhou, Jiangxi, China; 8 The Affiliated Ganzhou Hospital, Jiangxi Medical College, Nanchang University, Nanchang, China

**Keywords:** adrenal insufficiency, cystitis, diabetic ketoacidosis, gastric-type endocervical adenocarcinoma, immune checkpoint inhibitors, immune-related adverse events, rechallenge

## Abstract

**Background:**

Gastric-type endocervical adenocarcinoma (GEA) is an aggressive, HPV-independent malignancy with a poor prognosis. Cadonilimab, a bispecific antibody targeting PD-1 and CTLA-4, has shown promising efficacy in advanced cervical cancer but may increase the risk of immune-related adverse events (irAEs), including rare multiorgan involvement. Reports of sequential, multiorgan irAEs involving the urinary tract and endocrine system following cadonilimab therapy, as well as subsequent successful rechallenge, are exceedingly rare.

**Case presentation:**

A 70-year-old female with stage IVB GEA developed concurrent irAEs nine months after initiating first-line chemotherapy combined with cadonilimab and bevacizumab. She initially presented with urinary frequency, urgency, and fatigue. Investigations revealed grade II immune-related cystitis (based on imaging and persistently negative urine cultures) and concurrent grade III adrenal insufficiency (AI) with low serum cortisol and inappropriately normal ACTH levels. Immunotherapy was temporarily suspended, and she commenced hydrocortisone replacement therapy, resulting in symptom amelioration. After symptom resolution, cadonilimab was rechallenged on August 16, 2025. Four months later, she developed diabetic ketoacidosis (DKA) and received a diagnosis of grade IV immune-related diabetes mellitus (DM), requiring insulin therapy. After metabolic stabilization, cadonilimab was rechallenged again. As of the last follow-up, the patient had received 24 cycles of cadonilimab, achieving a progression-free survival (PFS) of 17 months with no recurrence of prior irAEs.

**Conclusion:**

This case highlights the rare but possible occurrence of sequential, multiorgan irAEs, including cystitis, AI, and DKA, following cadonilimab therapy. This case underscores the importance of multidisciplinary collaboration, vigilant monitoring, and individualized rechallenge decisions in managing complex irAEs. Importantly, it demonstrates that immunotherapy rechallenge can be feasible and provide sustained clinical benefit in selected patients with advanced GEA after resolution of severe irAEs.

## Introduction

First described in 2007, gastric-type endocervical adenocarcinoma (GEA) is the second most common cervical adenocarcinoma subtype (1, 2). Nearly all cases are HPV-negative (3, 4). GEA accounts for 10–29% of cervical adenocarcinomas in Asia-Pacific (2, 3, 5, 6). It frequently metastasizes to abdominal organs and the CNS, leading to advanced-stage diagnosis and a 5-year survival of only 42% (vs. 91% for conventional types) (6). Advanced GEA is treated with cisplatin/paclitaxel/bevacizumab, with or without immune checkpoint inhibitors (ICIs) for PD-L1-positive tumors (7).

Cadonilimab is a bispecific antibody targeting programmed cell death protein 1 (PD-1) and cytotoxic T lymphocyte-associated antigen 4 (CTLA-4) (8, 9). However, the enhanced immune activation conferred by dual PD-1/CTLA-4 blockade inherently increases the risk of immune-related adverse events (irAEs), particularly endocrinopathies. Meta-analyses have demonstrated that combination immunotherapy significantly elevates the incidence of all-grade endocrine irAEs compared to monotherapy (10, 11). Immune-related DM, though less common with an incidence of 0.4–1.9% (12–14), frequently presents as DKA and requires prompt recognition and intervention. Immune-related cystitis represents an exceedingly rare but increasingly recognized adverse event, with a recent single-center cohort reporting an incidence of approximately 0.96% among ICI-treated patients (15). Immune-related adrenal insufficiency (AI) often presents with nonspecific clinical manifestations (such as fatigue, anorexia, and malaise), which may be overlooked by oncologists in clinical practice.

Reports of synchronous or metachronous irAEs involving three or more organ systems (multiorgan) in a single patient are exceedingly rare. The objective of this case report is to describe the sequential development of three distinct irAEs—cystitis, adrenal insufficiency, and diabetic ketoacidosis—following cadonilimab therapy in a patient with advanced GEA, and to demonstrate that successful rechallenge is feasible after resolution of severe multiorgan irAEs. Furthermore, we discuss both the diagnostic and therapeutic challenges posed by irAEs affecting multiple organs.

## 2 Case presentation

### 2.1 Chief complaints

A 70-year-old female was admitted to the hospital on July 27, 2025, presenting with a one-week history of urinary frequency, urgency, fatigue, and fever.

### 2.2 Past medical history and family history

**Figure 1:**
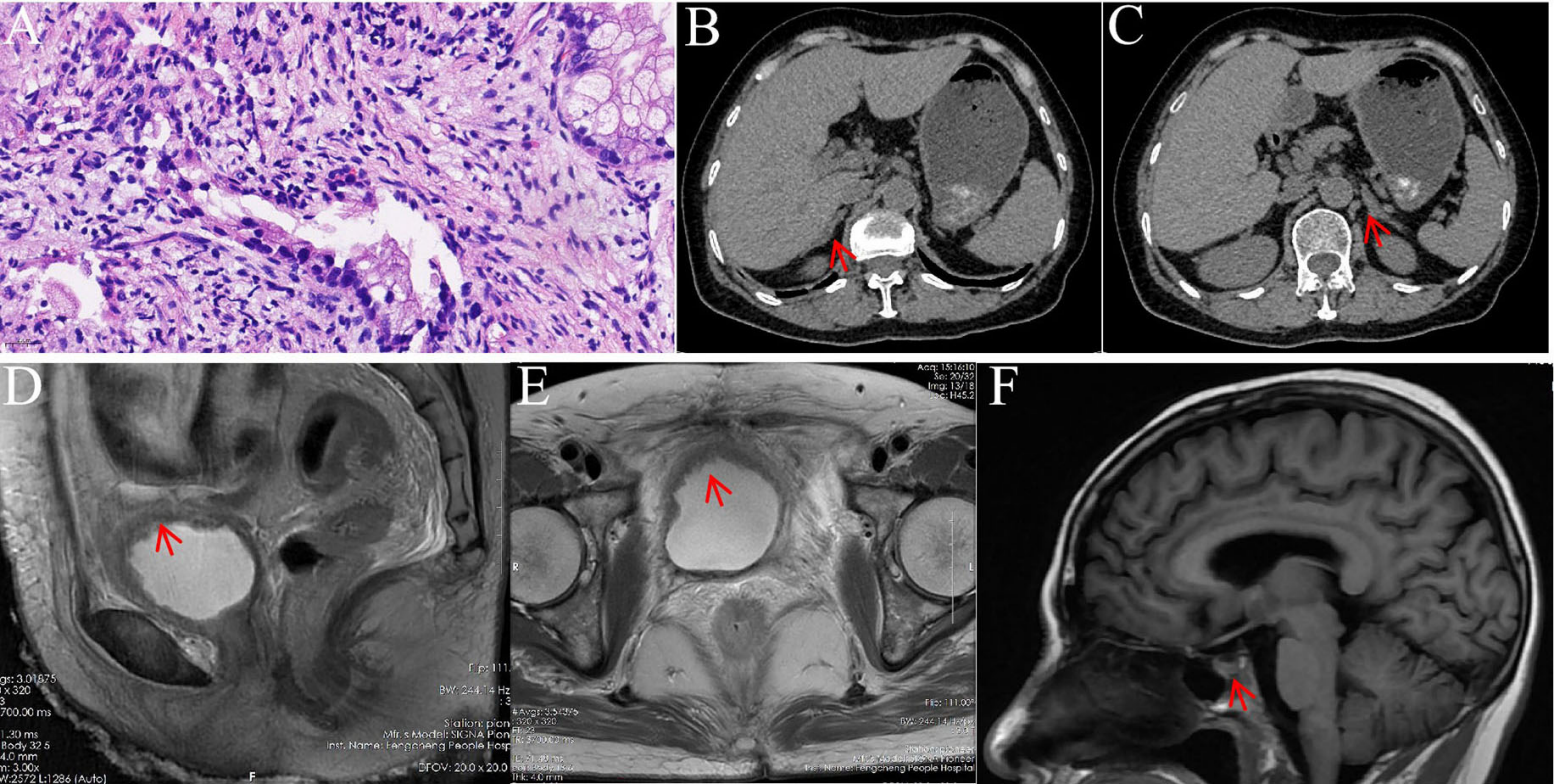
Pathological and imaging findings. **(a)** Depicts that Cervical biopsy showing GEA (HE, x400); **(b, c)** CT images of the right and left adrenal glands, respectively, demonstrating normal findings; **(d, e)** MRI images of the bladder in the sagittal and transverse planes, respectively, revealing diffuse and irregular wall thickening; **(f)** Sagittal MRI of the pituitary gland showing no abnormalities.

The patient had a prior hospitalization on October 11, 2024, during which she underwent a total hysterectomy with bilateral salpingo-oophorectomy, omentectomy, and bladder repair. Postoperative pathology confirmed gastric-type adenocarcinoma involving both ovaries, fallopian tubes, and the omentum (**Figure 1a**). Immunohistochemistry results were as follows: HER2 (0), TP53 (+), MSS, and PD-L1 expression in 70% of tumor cells. The final diagnosis at discharge was gastric-type adenocarcinoma of the cervix (Stage IVB). The patient subsequently completed six cycles of chemotherapy with paclitaxel (175 mg/m²), cisplatin (50 mg/m²), and cadonilimab (10 mg/kg) with bevacizumab (15 mg/kg, 6 weeks after the operation). This was followed by maintenance immunotherapy with cadonilimab plus bevacizumab every 21 days. The last maintenance treatment before this admission was given on July 8, 2025. The patient had received a total of 13 cycles of immunotherapy. She had no history of smoking or alcohol consumption and no notable family medical history.

### 2.3 Physical, laboratory, and imaging examinations

**Figure 2:**
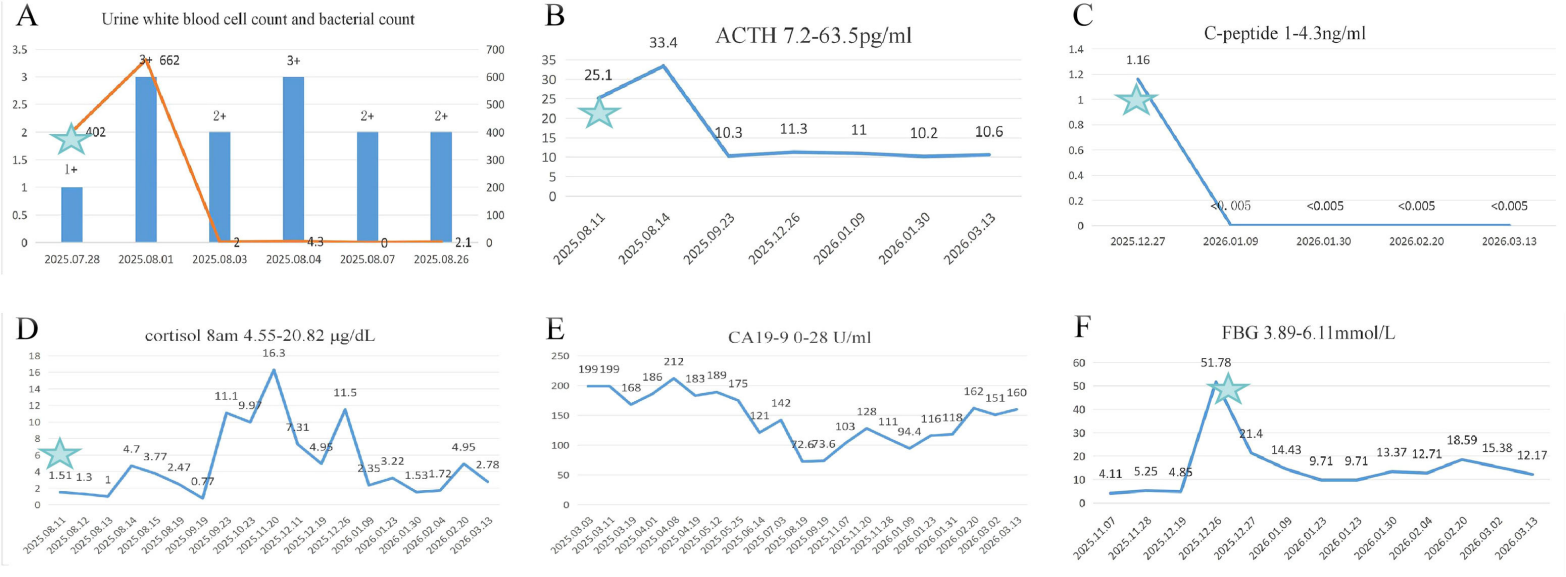
Laboratory values during the follow-up period. **(a)** ■: Urine white blood cell count and, _: Urine bacterial count, ★: Time of cystitis occurrence; **(b)** ACTH, ★: Time of AI occurrence; **(c)** C-peptide, ★: Time of DKA occurrence; **(d)** Cortisol 8am, ★: Time of AI occurrence; **(e)** CA19-9; **(F)** FBG, ★: Time of DKA occurrence.

Upon admission, the patient was hemodynamically stable with normal vital signs. Her Karnofsky Performance Status (KPS) score was 70, and her body mass index (BMI) was 20.4 kg/m². She reported voiding small volumes of urine every 20–30 minutes. Physical examination was otherwise unremarkable. Laboratory tests revealed serum cortisol levels of 1.51 μg/dL at 8:00 AM, 1.14 μg/dL at 4:00 PM, and 1.30 μg/dL at 11:00 PM. Adrenocorticotropic hormone (ACTH) level was 25.1 pg/mL. Serial urinalyses showed white blood cell levels ranging from 2+ to 3+ and bacterial counts of 200–400/μL (**Figure 2a**); however, repeated urine cultures remained negative, as did smears for tuberculosis and other bacterial pathogens. Liver and renal function panels, complete blood count, coagulation profile, cardiac enzymes, troponin, sex hormones (prolactin, testosterone, LH, progesterone, FSH, and estradiol), thyroid function, aldosterone, and rheumatologic autoimmune workup were all within normal limits. Chest and abdominal CT revealed no evidence of tumor recurrence or adrenal enlargement/atrophy (**Figures 1b, c**). The brain MRI was unremarkable (**Figure 1f**). Pelvic MRI demonstrated multiple bladder diverticula, diffuse bladder wall thickening, and dilation of the distal ureters, consistent with cystitis (**Figures 1d, e**).

### 2.4 Final diagnosis

The final diagnoses were: gastric-type adenocarcinoma of the cervix with liver and peritoneal lymph node metastases; grade II immune-related cystitis; grade III immune-related AI (graded according to CTCAE version 5.0 (16)).

### 2.5 Treatment

Following a multidisciplinary team discussion involving oncology, radiology, gynecology, urology, and endocrinology, maintenance therapy with cadonilimab and bevacizumab was temporarily suspended. The patient was started on hormone replacement therapy with hydrocortisone 20 mg twice daily, along with best supportive care. By August 12, her symptoms—including urinary frequency, urgency, muscle aches, and poor appetite—had significantly improved. During the follow-up period, the dosage was subsequently reduced by 5 mg every half month until it reached the physiological dose of 15 mg daily. On August 16, immunotherapy was rechallenged with cadonilimab, and maintenance therapy with bevacizumab was resumed.

### 2.6 Follow-up

After four months (August 16 to December 20, 2025) of maintenance immunotargeted therapy comprising seven cycles, the patient was readmitted on December 26, 2025, with complaints of dry mouth, generalized weakness, and urinary frequency. Fasting blood glucose (FBG) was measured at 51.78 mmol/L on laboratory examination, fasting C-peptide 1.16 ng/mL, and 2-hour postprandial C-peptide 1.11 ng/mL. Glycated hemoglobin (HbA1c) level was 6.69%. Arterial blood gas analysis showed pH 7.171, bicarbonate 5 mmol/L, base excess –21.9, anion gap 25.6 mmol/L, and lactate 3.18 mmol/L. Urinalysis revealed protein 2+, glucose 4+, ketones 2+, and pH 5.5. Anti-insulin antibodies, anti-islet cell antibodies, and anti-glutamic acid decarboxylase (GAD) antibodies were negative. Liver and kidney function, thyroid function, and complete blood count were within normal ranges. A diagnosis of Grade IV immune-related DKA was established. The patient received intravenous fluids, sodium bicarbonate for acidosis correction, potassium supplementation, and insulin therapy. After metabolic stabilization, she was discharged. On January 10, cadonilimab was rechallenged again, and bevacizumab maintenance was resumed. The last treatment cycle was administered on March 14. The patient received a total of 24 cycles of cadonilimab immunotherapy. According to RECIST v1.1 criteria, the best response was stable disease (SD), with a progression-free survival (PFS) of 17 months (**Supplementary Figure 6**). Immune-related DKA occurred after seven cycles following the first rechallenge. During follow-up, laboratory assessments revealed that ACTH levels remained within the normal range, while C-peptide was undetectable. Serum cortisol levels fluctuated between 1.53 and 16.3 μg/dL, and carbohydrate antigen 19-9 (CA19-9) ranged from 72.6 to 212 U/mL. Fasting blood glucose levels varied between 9.71 and 18.59 mmol/L (**Figures 2b-f**). As of the last follow-up on March 20, 2026, no recurrence of the above irAEs or new adverse events had been observed (**Supplementary Figure 3**).

## 3 Discussion

The sequential development of immune-related cystitis, AI, and DKA in this patient illustrates the broad spectrum of endocrine and non-endocrine toxicities associated with bispecific PD-1/CTLA-4 inhibition. Each individual irAE presents with its own unique epidemiological pattern, clinical manifestations, and diagnostic and therapeutic challenges, while their potential interactions warrant comprehensive consideration.

### 3.1 Current landscape of multiorgan irAEs

According to the literature, the majority of irAEs involve a single organ system. Available data suggest that the incidence of irAEs involving two organs ranges from 9.6% to 28.16% (17–19). Involvement of three or more organs in a single patient is rare; for instance, a comprehensive analysis of atezolizumab-treated non-small cell lung cancer patients found that irAEs affecting ≥3 organs accounted for only 1.92% (14/730) of all cases (18). This incidence may be underestimated in clinical practice due to lack of recognition.

In the present case, the patient sequentially developed three distinct irAEs—immune-related cystitis, AI, and DKA—following cadonilimab therapy. The timing of each event is noteworthy. Immune-related cystitis occurred nine months after ICI initiation, which is later than the median onset of 3–6 months reported in the literature (15). Concurrently, AI was diagnosed at the same time, a pattern rarely described. Immune-related DKA developed five months after AI and following the first rechallenge, with a fulminant progression from normoglycemia to DKA within days—consistent with the rapid β-cell destruction reported in 69.7% of ICI-related diabetes cases (20, 21). The seronegative autoantibody status (anti-GAD, anti-insulin, anti-islet cell) observed here aligns with the 40–60% seronegative phenotype in immune-related diabetes (22, 23). This pattern aligns with published observations that endocrine irAEs (e.g., AI, thyroiditis) often occur earlier (median 6–12 weeks), while diabetes may present later or after rechallenge (20, 23). Importantly, rechallenge can trigger new irAEs without reactivating prior ones, as seen here, suggesting distinct immunological targets and thresholds for different organs.

### 3.2 Diagnosis challenges of multiorgan irAEs

To date, no case of concomitant immune-related cystitis and AI has been reported, which complicates timely and accurate diagnosis in our patient. The initial presentation of lower urinary tract symptoms, along with fatigue and anorexia, posed a diagnostic challenge, as these symptoms closely mimic those of a common urinary tract infection—an alternative diagnosis that is often considered first. Since the first reported case of immune-related cystitis in 2017 (24), only 46 cases across 29 reports have been documented in the literature, underscoring its extreme rarity. Currently, there is no established gold standard for diagnosing immune-related cystitis. The diagnosis is primarily based on the combination of persistently negative urine cultures and imaging findings (CT or MRI) revealing bladder wall thickening with irregular margins, as seen in this case.

Because the patient received bevacizumab as maintenance therapy, other potential causes needed to be ruled out when making this diagnosis, including occult infection, bevacizumab toxicity, and bladder diverticulum. Occult infection was excluded by repeatedly negative urine cultures and no response to antibiotics. Bevacizumab-related urinary toxicity usually presents as hemorrhagic cystitis or proteinuria, whereas diffuse bladder wall thickening is extremely rare. Moreover, the patient’s symptoms completely resolved after immune checkpoint inhibitor (ICI) interruption, and bevacizumab was continued without recurrence. The presence of a bladder diverticulum does not explain the diffuse wall thickening or the symptom of urinary frequency.

The diagnosis of immune-related AI often remains incidental, owing to its nonspecific clinical manifestations. ICI-induced AI can be classified as primary (adrenalitis) or secondary (hypophysitis). Primary AI typically presents with low cortisol levels accompanied by elevated ACTH, whereas secondary AI is characterized by low cortisol with inappropriately low or normal ACTH levels (25)—a pattern consistent with the present case. ICI-related AI is most commonly secondary to hypophysitis. A retrospective study found pituitary enlargement in 94% (16/17) of patients who developed hypophysitis following combination therapy with ipilimumab and nivolumab (26). In contrast, our patient exhibited no evidence of pituitary or adrenal enlargement on imaging, which is atypical for hypophysitis induced by PD-1/CTLA-4 bispecific antibody therapy. Nevertheless, isolated impairment of corticotroph function without pituitary enlargement has also been reported (23, 27, 28). Consequently, the Society for Immunotherapy of Cancer (SITC) Toxicity Management Working Group recommends baseline and periodic monitoring of cortisol and ACTH levels in patients receiving ICIs, particularly when symptoms suggestive of AI emerge (29).

While the diagnosis of DKA in this patient was unequivocal, its etiology required careful differentiation between immune-related DKA and corticosteroid-induced DKA, given that hyperglycemia emerged following glucocorticoid treatment for immune-related cystitis and AI. A retrospective study of cancer patients with DKA found that 28.6% (26/84) of cases were attributable to ICIs, whereas 11% were associated with high-dose corticosteroid therapy (30). A mixed mechanism is possible: corticosteroid-induced insulin resistance may have contributed to hyperglycemia, but the rapid decline in C-peptide to undetectable levels and the need for lifelong insulin point to autoimmune β-cell destruction as the primary driver. Fulminant type 1 diabetes (FT1D), which is more common in East Asian populations, should be considered in the differential diagnosis; the modestly elevated HbA1c (6.69%) despite rapid onset of DKA is characteristic of FT1D, reflecting insufficient time for glycation. In the present case, the patient had no prior history of diabetes and tested negative for pancreatic islet autoantibodies. Following the onset of DKA, serum C-peptide levels declined rapidly and became nearly undetectable within 13 days, accompanied by only a modest elevation in hemoglobin A1c. Collectively, these findings are consistent with the diagnostic criteria for immune-related DM (12).

### 3.3 Treatment of multiorgan irAEs

The management of multiorgan irAEs necessitates a coordinated, multidisciplinary approach. For immune-related cystitis presenting with lower urinary tract symptoms that impair quality of life, no standardized treatment paradigm currently exists. Corticosteroids remain a mainstay of therapy; however, optimal initial dosing and duration of treatment are matters of debate. A single-center retrospective study reported that most patients received prednisone at an initial dose of 30–60 mg/day, followed by a gradual taper over 6 to 10 weeks (15), representing the first study to propose a specific dosing and tapering schedule. Nevertheless, disease recurrence has been observed during corticosteroid tapering in some cases.

According to current ESMO guidelines, the management of isolated AI secondary to hypophysitis centers on hormone replacement therapy, typically with hydrocortisone (20–25 mg/day or 10–12 mg/m²) or prednisolone (5–7.5 mg/day) (25, 31). Following symptom resolution, long-term physiological maintenance therapy (e.g., hydrocortisone 10–20 mg/day) is recommended. Previous studies indicate that high-dose corticosteroids (e.g., prednisone 1 mg/kg/day or equivalent) do not contribute to the recovery of pituitary function (32) and may lead to shorter survival compared with low-dose regimens (23, 33); therefore, their use is limited to patients presenting with clinically significant manifestations.

In the present case, the patient received guideline-directed corticosteroid therapy (hydrocortisone 20 mg twice daily), which led to significant improvement in urinary symptoms, fatigue, and anorexia. The dose was successfully tapered by 5 mg every two weeks. However, in contrast to most reported cases, corticosteroid therapy was not completely discontinued in this patient due to concurrent immune-related AI; instead, physiological maintenance therapy (hydrocortisone 15 mg/day) was continued. During follow-up, no recurrence of cystitis or AI was observed.

The emergency management of immune-related DKA should follow the measures outlined in current guidelines, which include fluid resuscitation, insulin administration, and correction of electrolyte imbalances (34). Given the irreversible loss of pancreatic β-cell function, lifelong insulin replacement therapy is necessary (35). Following appropriate acute management, symptoms of DKA typically improve or resolve within one week (36), as observed in the present case. However, insulin management in patients with concurrent AI requires careful coordination. Given that glucocorticoid replacement therapy significantly affects insulin sensitivity, dose adjustments of either treatment must take their complex interaction into account. Patient education on sick-day management, including stress dosing of hydrocortisone during illness and glucose monitoring during steroid adjustments, is essential for preventing recurrent metabolic crises and adrenal crises.

### 3.4 Management of immune rechallenge for multiorgan irAEs

The decision to rechallenge with ICIs following significant toxicity remains one of the most contentious and clinically challenging scenarios in modern oncology, particularly in patients who have experienced three or more irAEs. To date, no clinical studies have specifically evaluated the risk-benefit ratio of immunotherapy rechallenge after multiorgan irAEs.

Among the 18 case reports on multiorgan irAEs (**Table 1**) (37–54), six occurred following combination therapy with ipilimumab and nivolumab; notably, no cases have been reported with cadonilimab. Among these 18 patients, seven underwent ICI rechallenge. Of these seven patients, only one experienced no recurrent irAEs (37), while six developed new-onset irAEs. Specifically, one patient discontinued therapy after developing hypothyroidism and AI following the first rechallenge (38); one patient treated with ipilimumab plus nivolumab developed immune-related DKA following two rechallenges and completion of therapy (39); one patient with severe multiorgan irAEs resumed treatment with a reduced dose of pembrolizumab after a 10-month washout, achieving favorable tumor control with only mild recurrence of irAEs (40); one patient underwent three rechallenges with sustained disease control (41); and another patient developed severe DKA with AI after the first rechallenge, then received a second rechallenge with nivolumab alone after symptom stabilization (42). Importantly, no fatal adverse events were reported among any of these rechallenged patients.

**Table 1:** Summary of case report of multiorgan irAEs (involving ≥3 organs) and outcomes of ICI rechallenge.

Case	Primary cancer	Therapy	irAEs	Immune rechallenge	Process of rechallenge
Sum M (2018) (44)	Melanoma	Nivo + Ipi	DM, Hypophysitis, Hypothyroidism	No	
Gunjur A (2019) (39)	Melanoma	Pem	DM, AI, Hypothyroidism	Yes	DKA after C1 → (First Rechallenge) Hypothyroidism and AI developed → Treatment stopped
Khalid M (2019) (40)	Melanoma	Nivo + Ipi	Hypothyroidism, AI, DM	Yes	Hypothyroidism after C4 → (First R) AI occurred on nivolumab → (Second R) Nivo was continued for 1 year then discontinued; DKA occurred after treatment cessation
Lanzolla G (2019) (45)	Lung adenocarcinoma	Atezo	DM, PAI, Hypophysitis	No	
Marshall S (2020) (46)	Gastric Cancer	Nivo	DM, Hypophysitis, Hypothyroidism	No	
Shen L (2021) (41)	Thymic epithelial tumors	Pem	Liver and Kidney dysfunction, Hypothyroidism, myocarditis	Yes	Hepatorenal dysfunction, hypothyroidism, and myocarditis after C1; treatment stopped → After 10 months, (First R) a half-dose rechallenge led to mild recurrence of irAEs and tumor shrinkage
Chen Y (2022) (47)	Mixed liposarcoma	Tori	Myocarditis, Myositis, Thyroiditis, Skin toxicity	No	
Hino C (2022) (42)	Sarcomatoid renal cell carcinoma	Nivo + Ipi	DM, AI, Hypothyroidism, Acute interstitial nephritis	Yes	Hypothyroidism after 2m → (First R) Interstitial nephritis at 4m → (Second R) DM developed at 8m → (Third R) Hypophysitis at 10m on maintenance Nivo
Fuentes-Antrás J (2022) (48)	Lung Adenocarcinoma	Pem	Thyroiditis, hepatitis, myositis, myocarditis, pneumonitis	No	
Takata M (2022) (49)	Melanoma	Nivo	Thyroiditis, Hypophysitis, DM	Yes	Hypothyroidism after C3 → (First R) AI after C7 → (Second R) DKA after C18
Ishiguro A (2022) (38)	Melanoma	Nivo	DM, Thyroiditis, Hypophysitis	Yes	(First R) No irAEs observed
Luo J (2022) (50)	Non-small cell lung cancer	Pem	Hypothyroidism, DKA, Hypophysitis	No	
Iesaka H (2023) (43)	Metastatic renal cell carcinoma	Nivo + Ipi	Thyroiditis, DM, Hypophysitis	Yes	Thyroiditis after C2 → (First R) DKA with AI occurred after C4; treatment stopped → (Second R) After symptom stabilization, nivolumab maintenance was resumed
Tayyeb M (2023) (51)	Breast cancer	Pem	PAI, DKA, Hypothyroidism	No	
Luong B (2024) (52)	Metastatic pancreatic adenocarcinoma	Pem	DKA, Enteritis, Thrombotic Thrombocytopenic Purpura (TTP)	No	
Janulevicius T (2025) (53)	Melanoma	Pem	Myocarditis, DKA, CNS Demyelination	No	
Nur AM (2025) (54)	Melanoma	Nivo + Ipi	DKA, Myocarditis, AI	NR	
Vengilote R (2026) (55)	Metastatic renal cell carcinoma	Nivo + Ipi	Hypothyroidism, DM, AI, Inflammatory arthritis	No	

Atezo, atezolizumab; Ipi, ipilimumab; Nivo, nivolumab; Pem, pembrolizumab; Tori, toripalimab; DM, diabetes mellitus; AI, adrenal insufficiency; DKA, diabetic ketoacidosis; PAI, primary AI; CNS, central nervous system; C, cycle; R, rechallenge; m, month; NR, not reported.

According to several clinical guidelines, the presence of endocrine irAEs does not constitute an absolute contraindication for the continuation of ICI therapy. If patients are stabilized on hormone or insulin replacement therapy, ICI treatment can generally be safely resumed (55–58), particularly in those who have demonstrated a favorable tumor response (59). In a case series of immune-related cystitis, only four patients underwent rechallenge, of whom two experienced no recurrence of adverse effects.

Given the poor prognosis of advanced GEA and the limited therapeutic options available, we weighed the potential clinical benefit against the manageable toxicity profile and elected to proceed with cadonilimab rechallenge after symptom control. Following the first rechallenge, the patient developed immune-related DKA after four months of treatment. After aggressive management and symptom stabilization, we opted to continue cadonilimab for a second rechallenge without dose reduction or switch to alternative therapy. As of March 20, 2026, no new adverse events have occurred, and the tumor assessment indicates stable disease.

To our knowledge, no prior reports have described a patient with GEA who developed sequential immune-related cystitis, DKA, and AI following cadonilimab therapy and subsequently underwent successful rechallenge with the same immunotherapeutic agent. Several limitations should be acknowledged. First, this is a single case report, and findings may not be generalizable. Second, anti-GAD65 antibody was not measured. Third, formal HPA axis recovery testing was not performed. Fourth, we did not perform bladder biopsy to histopathologically confirm immune-related cystitis. Fifth, ACTH stimulation test was not performed. Despite these limitations, this case provides valuable real-world evidence for managing complex, multiorgan irAEs and supports the feasibility of rechallenge in selected patients.

## 4 Conclusions

This case highlights the rare but possible occurrence of multiorgan irAEs following cadonilimab therapy. Prompt recognition, multidisciplinary collaboration, and individualized rechallenge enabled sustained clinical benefit with manageable toxicities. Close follow-up facilitated early detection of subsequent irAEs, allowing timely intervention and continued immunotherapy. This case underscores the importance of vigilance, interdisciplinary communication, and patient-centered decision-making in optimizing outcomes for patients experiencing complex irAEs.

## Data Availability

The original data presented in the study are included in the article or **Supplementary Material**. Further inquiries can be directed to the corresponding authors.
