# Supplementary material for: Case Report: Successful rechallenge of cadonilimab-induced cystitis, adrenal insufficiency, and diabetic ketoacidosis in a patient with metastatic gastric-type endocervical adenocarcinoma
Source: Front Oncol. 2026 May 20;16:1836563. doi: 10.3389/fonc.2026.1836563 (PMC13229985; doi:10.3389/fonc.2026.1836563)
Supplement: Supplementary file 2 [file DataSheet2.docx]

****Postoperative Pathological Report****

****Diagnosis:**** Gastric-type adenocarcinoma of the uterus (non-HPV-associated adenocarcinoma), moderately differentiated.

****Immunohistochemistry:**** MLH (+), MSH6 (+), HER2 (0), P53 (+), Syn (-), PAX8 (+), ER (-), PR (-), P63 (-), CK7 (+), CK20 (-), CDX-2 (partial +), MUC6 (partial +), TTF-1 (-), GATA3 (-), villin (+), IMP3 (partial +).

****PD-L1 Testing Result:**** Combined Positive Score (CPS) for PD-L1 expression on tumor cells and immune cells: 70.

****Notes:**** Antibody clone: E1L3N; Detection platform: Leica BOND-MAX automated stainer; Quality control: qualified; Tumor cell count: > 100.
