# Supplementary material for: Case Report: Successful rechallenge of cadonilimab-induced cystitis, adrenal insufficiency, and diabetic ketoacidosis in a patient with metastatic gastric-type endocervical adenocarcinoma
Source: Front Oncol. 2026 May 20;16:1836563. doi: 10.3389/fonc.2026.1836563 (PMC13229985; doi:10.3389/fonc.2026.1836563)
Supplement: Supplementary file 3 [file DataSheet3.docx]

**Supplementary materials Figure legends**


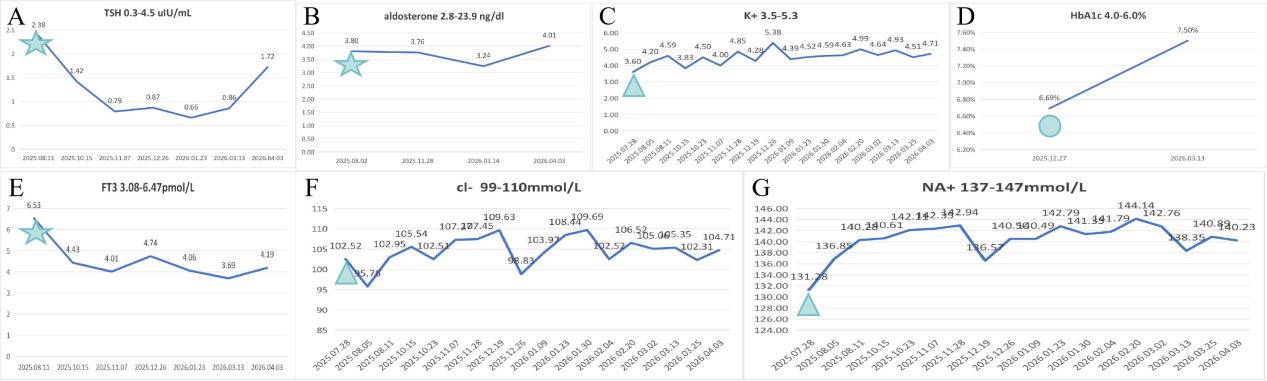


Figure 3 Other laboratory values during the follow-up period. (a) TSH**; (b) aldosterone; (c)** K+**; (d) HbA1c; (e) FT3; (F)** cl-**; (g) NA+.** ★: **Time of AI occurrence; ▲: Time of c**ystitis **occurrence; ●: Time of DKA occurrence.**


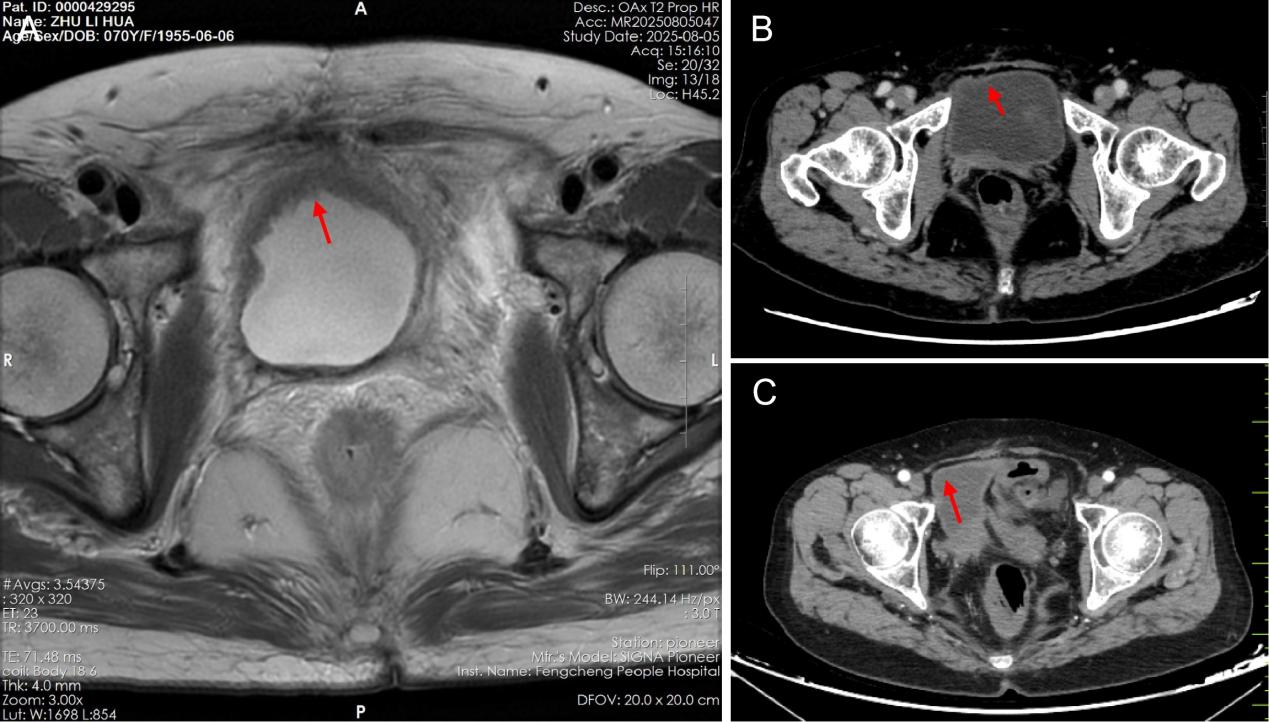


Figure 4 Imaging findings of the bladder wall during follow-up. (a) MRI images of the bladder in the transverse planes, respectively, revealing diffuse and irregular wall thickening (August 5, 2025); (b, c) **CT shows a normal bladder wall (November 28, 2025; April 3, 2026).**

**
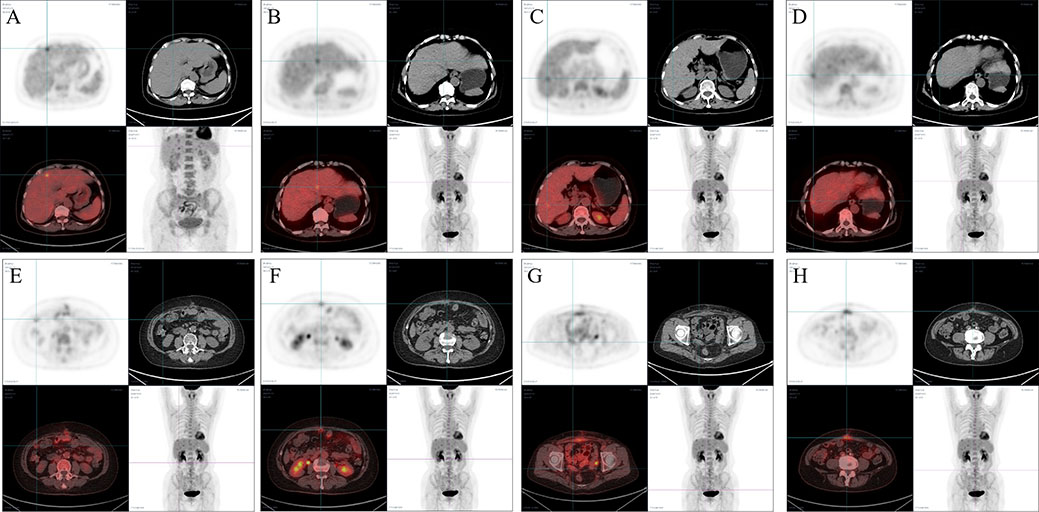
**

Figure 5 **PET-CT imaging findings from November 15, 2024.** (A, B) Metastatic lesion in the left lobe of the liver (lesion 1, 2); (C, D) Metastatic lesion in the right lobe of the liver (lesion 1, 2); (E, F, G) Peritoneal metastatic lesion (lesion 1, 2, 3); (H) Abdominal wall metastatic lesion.

**
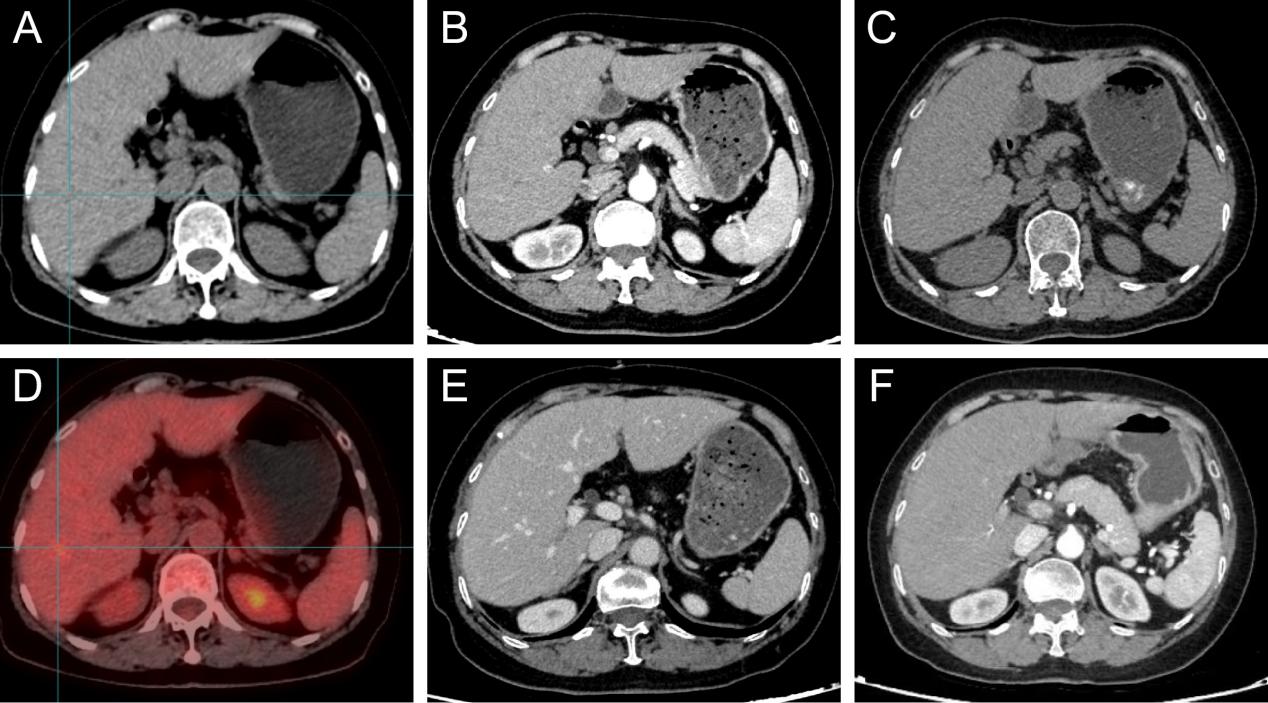
**

**Figure 6 Therapeutic response assessment on abdominal CT during follow-up. (a, d) PET‑CT on November 15, 2024, showing a metastatic nodule in the right lobe of the liver; (b, c, e, f) CT of the right liver lobe on March 3, 2025, August 1, 2025, November 28, 2025, and April 3, 2026.**
